# PACE-UP (Pedometer and consultation evaluation - UP) – a pedometer-based walking intervention with and without practice nurse support in primary care patients aged 45–75 years: study protocol for a randomised controlled trial

**DOI:** 10.1186/1745-6215-14-418

**Published:** 2013-12-05

**Authors:** Tess Harris, Sally M Kerry, Christina R Victor, Sunil M Shah, Steve Iliffe, Michael Ussher, Ulf Ekelund, Julia Fox-Rushby, Peter Whincup, Lee David, Debbie Brewin, Judith Ibison, Stephen DeWilde, Elizabeth Limb, Nana Anokye, Cheryl Furness, Emma Howard, Rebecca Dale, Derek G Cook

**Affiliations:** 1 Population Health Research Centre, St George’s University of London, London SW17 ORE, UK; 2 Pragmatic Clinical Trials Unit, Queen Mary’s University of London, London E12AT, UK; 3 Gerontology and Health Services Research Unit, Brunel University, London UB8 3PH, UK; 4 Department of Population Health Sciences, University College, London NW3 2PF, UK; 5 MRC Epidemiology Unit, University of Cambridge, Cambridge CB2 OQQ, UK; 6 Department of Sport Medicine, Norwegian School of Sport Sciences, PO Box 4014, 0806 Oslo, Norway; 7 Health Economics Research Group, Brunel University, London UB83PH, UK; 8 10 Minute CBT, Devonshire Business Centre, Letchworth Garden City, Herts SG61GJ, UK

**Keywords:** Accelerometers, Behaviour change techniques, Cognitive behavioural, Middle-aged adults, Older people, Pedometers, Physical activity, Postal, Practice nurse, Primary care, Walking intervention

## Abstract

**Background:**

Most adults do not achieve the 150 minutes weekly of at least moderate intensity activity recommended for health. Adults’ most common physical activity (PA) is walking, light intensity if strolling, moderate if brisker. Pedometers can increase walking; however, most trials have been short-term, have combined pedometer and support effects, and have not reported PA intensity. This trial will investigate whether pedometers, with or without nurse support, can help less active 45–75 year olds to increase their PA over 12 months.

**Methods/design:**

*Design:* Primary care-based 3-arm randomized controlled trial with 12-month follow-up and health economic and qualitative evaluations.

*Participants:* Less active 45–75 year olds (n = 993) will be recruited by post from six South West London general practices, maximum of two per household and households randomised into three groups. Step-count and time spent at different PA intensities will be assessed for 7 days at baseline, 3 and 12 months by accelerometer. Questionnaires and anthropometric assessments will be completed.

*Intervention:* The pedometer-alone group will be posted a pedometer (Yamax Digi-Walker SW-200), handbook and diary detailing a 12-week pedometer-based walking programme, using targets from their baseline assessment. The pedometer-plus-support group will additionally receive three practice nurse PA consultations. The handbook, diary and consultations include behaviour change techniques (e.g., self-monitoring, goal-setting, relapse prevention planning). The control group will receive usual care.

*Outcomes:* Changes in average daily step-count (primary outcome), time spent sedentary and in at least moderate intensity PA weekly at 12 months, measured by accelerometry. Other outcomes include change in body mass index, body fat, self-reported PA, quality of life, mood and adverse events. Cost-effectiveness will be assessed by the incremental cost of the intervention to the National Health Service and incremental cost per change in step-count and per quality adjusted life year. Qualitative evaluations will explore reasons for trial non-participation and the interventions’ acceptability.

**Discussion:**

The PACE-UP trial will determine the effectiveness and cost-effectiveness of a pedometer-based walking intervention delivered by post or practice nurse to less active primary care patients aged 45–75 years old. Approaches to minimise bias and challenges anticipated in delivery will be discussed.

**Trial registration:**

ISRCTN98538934

## Background

### Benefit and risks of PA and current PA guidelines

*Why is physical activity (PA) important for adults and older adults?* PA leads to reduced mortality, a reduced risk of over 20 diseases and conditions, and improved function, quality of life and emotional well-being [1]. Physical inactivity is the fourth leading risk factor for global mortality [2] and a major cost burden on health services [1].

*What are the PA guidelines?* Adults and older adults are advised to be active daily and, in order to obtain health benefits, should achieve at least 150 minutes (2 ½ hours) per week of at least moderate intensity activity in bouts of 10 minutes or more. One effective way to do this is by 30 minutes of moderate intensity activity on at least 5 days weekly [1,3,4]. Regular walking is the most common PA of adults and older adults, walking at a moderate pace (3 mph /5 km/h) qualifies as moderate intensity PA [5]. Time spent being sedentary for extended periods should also be minimised, as this is an independent disease risk factor [1] and increases steeply from the age of 45 [6]. Whilst amongst adults in England aged 16 and over, 39% of men and 29% of women were judged to meet the recommended PA levels, based on their self-reported data, only 20% and 17% of men and women aged 60–74 met recommended levels [6], despite most of these inactive older people being capable of walking [7]*.* Lower socioeconomic groups [6] and Indian, Pakistani, Bangladeshi and Chinese ethnic groups are significantly less likely to report activity levels that meet the recommended levels, whilst the activity levels of other ethnic groups (Black Caribbean, Black African and Irish) are similar to that of the general population [8]. Surveys of adults in Europe and the USA also confirm that over 50% do not achieve public health PA recommendations [9,10]. Since PA, including walking, is unreliably recalled, surveys may overestimate PA levels [11]. Objective accelerometer measurement found that only 5% of men and 4% of women aged 35–64 years and 5% men and 0% of women aged 65 or more achieved the recommended PA levels, only a fraction of those self-reporting achieving these levels [6].

*What are the risks from increasing PA?* Risks from a sedentary lifestyle far exceed the risks from regular PA [3,12,13]. Moderate intensity PA carries a low injury risk [14], mainly musculoskeletal injury or falls [15]. Walking is very low risk, “a near perfect exercise” [5]. Screening participants for contraindications before participating in light to moderate intensity PA programmes is no longer advocated [3,16]. An important safety feature of our study is that individualised goals can be set from the participant’s own baseline, in line with advice that older adults in particular should start with low intensity PA and increase intensity gradually, the “start-low-and-go-slow” approach [12,13].

### Strategies for increasing PA

*How can adults and older adults increase their PA levels?* A systematic review of PA interventions reported moderate positive short-term effects, but findings were limited by mainly unreliable self-report measures in motivated volunteers [17]. Effective interventions explored factors associated with behavioural change, including beliefs about costs and benefits of PA [18]. Exercise programs in diverse populations can promote short- to medium-term increases in PA when interventions are based on health behaviour theoretical constructs, individually tailored with personalised activity goals and use behavioural strategies [3,19]. A critical review and a best practices statement on older peoples’ PA interventions advised home rather than gym-based programmes and behavioural strategies (e.g., goal-setting, self-monitoring, self-efficacy, support, relapse prevention training) rather than health education alone [13,20]. National Institute for Health and Clinical Excellence (NICE) guidance concluded that no particular behaviour change model was superior and that training should focus on generic competencies and skills rather than specific models [21]. Starting low, but gradually increasing to moderate intensity is promoted as best practice, with advice to incorporate interventions into the daily routine (e.g., walking) [13]. A recent systematic review concluded that walking interventions tailored to people’s needs, targeted at the most sedentary and delivered at the individual or household level, can be effective, although evidence directly comparing interventions targeted at individuals, couples or households is lacking [22].

*Are pedometers helpful?* Pedometers are small, inexpensive devices, worn at the hip, that provide direct step-count feedback. A systematic review of 26 studies found pedometer users increased steps/day by 2,491 (1,098–3,885) and PA levels by 27%, with significant reductions in body mass index (BMI) and blood pressure [23]. A second review (32 studies) found an average increase of 2,000 steps/day for pedometer users [24]. Step-goals and diaries were key motivational factors [23,24]. Several limitations were recognised. Study sizes were relatively small and long-term effects undetermined; many included several components (e.g., pedometer and support) so independent effects were difficult to establish and the inclusion of older people and men was very limited [23,24]. Recent studies have addressed some of these limitations. A trial of 210 older women found that a pedometer plus behaviour change intervention increased PA at 3 months but not at 6 months [25]. Two trials in high risk groups (cardiac disease and impaired glucose tolerance) showed sustained increases in step-count at 12 months [26,27]. NICE recently updated its advice from only advising pedometers as part of research [28] to now advising their use as part of packages including support to set realistic goals, monitoring and feedback [29].

*How do step-count goals relate to PA recommendations?* Step-count goals lead to more effective interventions, but no specific approach to goal-setting is favoured [23]. Goals are based on either a fixed target (e.g., 10,000 steps/day) [30,31] or by advising incremental increases on baseline, as a percentage (5% per week [32], 10% biweekly [33] or 20% monthly [25]) or by a fixed number of extra steps. Those advocating a fixed number of extra daily steps have developed step-based guidelines to fit with existing evidence based guidelines with their emphasis on 30 minutes of at least moderate intensity PA on 5 or more days weekly [34]. Despite individual variation, moderate intensity walking appears approximately equal to at least 100 steps per minute [34,35]. Multiplied by 30 minutes this produces a minimum of 3,000 steps per day, to be done over and above habitual activity. Several studies have advocated adding in 3,000 steps/day on most days weekly, either from the beginning [26] or by increasing incrementally (initially an extra 1,500 steps/day and increasing) [36,37] or increasing by 500 steps/day biweekly [27]. Studies that advised adding 3,000 steps/day to baseline produced significant improvements in step-counts at 3 months and two measured outcomes at 12 months and showed sustained improvements in step-counts [26,27], waist circumference [26] and fasting glucose levels [27]. Although there is no evidence at present to inform a moderate intensity cadence (steps/minute) in older adults, Tudor-Locke et al. advocate using the adult cadence of 100 steps/minute in older adults (whilst recognising that this may be unobtainable for some individuals) and advise that the 30 minutes can be broken down into bouts of at least 10 minutes [38]. This model was used in a primary care walking intervention in 41 older people which found significant step-count increases from baseline to week 12, maintained at week 24 [39,40].

*Could accelerometers be useful in a pedometer-based walking intervention?* Accelerometers are small activity monitors, worn like pedometers, more expensive, but able to provide a time-stamped record of PA frequency (step-counts) and intensity (activity counts). They require computer analysis and give no immediate feedback, functioning as blinded pedometers in objectively measuring baseline and outcome data, but providing objective data on time spent in different PA intensities, including time spent in at least moderate intensity activity and time spent sedentary, two important public health outcomes. Pedometer studies without accelerometers have relied on self-report measures of these outcomes. Accelerometers are valid and acceptable to adults [6,41] and older adults [6,42,43]. Although both instruments measure step-count and are highly correlated [44,45], pedometers usually record lower step-counts, particularly at lower walking speeds, and accelerometers cannot reliably be substituted for pedometers at an individual level [45]. Thus, although we will use the accelerometer to measure outcomes, we will use a blinded pedometer, worn simultaneously at baseline, to set individual step-count targets.

*Are pedometers cost-effective?* There is limited knowledge on the cost-effectiveness of pedometer-based interventions in the UK. Recent systematic reviews that considered the economic outcomes of pedometer-based interventions found no evidence [46,47], partly attributable to insufficient data [48]. However, a recent study assessed the cost-effectiveness of giving an individualised walking programme and pedometer with or without a consultation compared with usual walking activity alongside a trial of 79 people [49]. The incremental cost-effectiveness ratios per person achieving an additional 15,000 steps/week were £591 and £92 with and without the consultation. However, no data on quality of life were collected and impacts on long-term outcomes were not estimated.

*What is primary care’s role in promoting PA?* Primary care centres (general practices) in the UK provide healthcare and health promotion free at the point of access, to a registered list of local patients, using disease registers to provide annual or more frequent review of chronic disorders (for many of which PA will be of benefit), via a multi-disciplinary health care team to provide continuity of care. NICE guidance found that brief interventions in primary care are cost-effective and therefore recommends that all primary care practitioners should take the opportunity, whenever possible, to identify inactive adults and provide advice on increasing PA levels [28]. New National Health Service health checks include adults up to age 74 and incorporate advice on increasing PA, often by primary care nurses [50]. Primary care nurses have been shown to be effective at increasing PA, particularly walking, in this age group [51]. Health professional PA advice in consultations is individually tailored [52] and has greater impact than other PA advice [53]. PA promotion by other routes, for older adults in particular, is unlikely to be as effective [54]]. Exercise prescribing guidance in primary care reinforces the importance of follow-up to chart progress, set goals, solve problems, and identify and use social support [55]; this will be an important feature of the nurse PA consultations in this trial. Evaluation of the UK Step-O-Meter Programme, delivering pedometers through primary care, showed self-reported PA increases, but advised investigation with a RCT design [36]. Two small trials have assessed the effectiveness of pedometers plus PA consultations: one showed a significant effect on step-counts at 12 weeks in 79 middle-aged adults [37]; the other showed a significant effect on step-counts at 12 weeks, maintained at 24 weeks in 41 older primary care patients and called for a further, larger primary care trial [39,40].

*Theory on which the intervention is based and relevant pilot and preparatory work.* The pedometer-based intervention is centred on work cited above showing that pedometers can increase step-counts and PA intensity [23,24], but extending this to ensure that the study covers older adults, men, has a 12 month follow-up, and is designed to examine pedometer and support components separately. The patient handbook, diary and practice nurse PA consultations will use behaviour change techniques (BCTs) (e.g., goal-setting, self-monitoring, feedback, boosting motivation, encouraging social support, addressing barriers, relapse anticipation etc.). These techniques have been successfully used by non-specialists in primary care after brief training [56] and are emphasized in the Health Trainer Handbook [57], based on evidence from a range of psychological methods and intended for National Health Service behaviour change programmes, with local adaptation [57]. We have adapted the Health Trainer Handbook for use in this trial into PACE-UP nurse and patient handbooks, to focus specifically on PA using pedometers. The BCTs have been classified according to Michie’s refined taxonomy of BCTs for PA interventions [58] (Tables 1 and 2). Diary recording of pedometer step-counts provides clear material for PA goal setting, self-monitoring and feedback, and should fit well with this approach. Relevant pilot and preparatory work includes observational work using pedometers and accelerometers in primary care [42] and a trial with older primary care patients developing the PA consultations and pedometer-based walking intervention (PACE-Lift trial ISRCTN42122561) [59].

**Table 1:** PACE-UP patient handbook and diary, and behavioural change techniques included

	**Guide to content**	**Behavioural change techniques **[58]
Patient handbook	Health benefits of increasing walking	1, 2
	PA guidelines	4
	Moderate intensity PA and relating it to number of steps	
	PACE-UP walking programme and step-count targets	7,9,16
	Review participant baseline step-count	19
	How to increase PA safely	21
	Useful websites	4
	How to keep going when PACE-UP programme finishes	1,2,16,26,29,35
Patient diary	How to use pedometer and record steps in diary	16, 21
	Frequently asked questions on PACE-UP trial	
	Weekly recording of step-count and walking in diary (weeks 1–12)	7,9,19,26
	Achievement of targets (weeks 1–12)	10,12,13
	Planning when to walk, where to walk, who to walk with	20,29
	Week 2 Tips and motivators: make walking part of your daily routine	20
	Week 3 Ttips and motivators: remember personal benefits, what to do if you	2,20,35
	are falling behind your targets	
	Week 4 Keep it up: praise and reward yourself, encouraging social support	12,13,29
	Week 5 Keep motivated: write down step-counts, ask for support	12,16,29
	Week 6 Now we are moving: obstacles and solutions	8
	Week 7 How to make these changes permanent – ideas for new walks, making time for walking, what gains have been made so far?	38,17,11
	Week 8 Maintain the gain: pacing, tips for safe exercising	9,21,35
	Week 9 Be busy being active: keep monitoring with pedometer, places, people and thoughts that motivate you	16,29,36
	Week 10 Change does not happen in a straight line! Preparing for setbacks	8,35
	Week 11 Make it a healthy habit: building regular exercise habits, creating if-then plans	1,2,7,23
	Week 12 I’ve changed: how to keep up your walking programme	16,20,29
	Congratulations you have completed the programme	11,16,17
	How to keep going when PACE-UP programme finishes	1,16,29

1. Provide general information on behaviour-health link; 2. Provide information on consequences to individual; 4. Provide normative information about others’ behaviour; 7. Action planning; 8. Barrier identification; 9. Set graded tasks; 10. Prompt review of behavioural goals; 11. Prompt review of outcome goals; 12. Prompt rewards contingent on effort; 13. Prompt rewards contingent on successful behaviour; 16. Prompt self-monitoring of behaviour; 17. Prompting self-monitoring of behavioural outcome; 19. Provide feedback on performance; 20. Provide information on when and where to perform the behaviour; 21. Provide instructions on how to perform the behaviour; 23. Teach to use prompts/cues; 26. Prompt practice; 29. Plan social support/social change; 35. Relapse prevention/coping planning; 36. Stress management/emotional control training; 38. Time management.

**Table 2:** PACE-UP practice nurse physical activity consultations and behaviour change techniques included

**Week**	**Sessions**	**Guide to session content**	**Behavioural change techniques **[58]
1	Session 1: First steps (30 minutes) Week 1	Review health status, current activity, health benefits of PA	1, 2
		Cost-benefit analysis for increasing PA	2
		PA guidelines and how to increase PA safely	4, 21
		Moderate intensity PA and relating it to number of steps	
		Review participant baseline step-count	19,
		Teach use of pedometer and recording walks and steps in diary	21, 26
		Ideas for increasing steps	20
		Goal-setting – PACE-UP goals or tailored to the individual patient	7, 9, 16
		Use of rewards for effort and for achieving goals	12, 13
		Summarise and check patient understanding, plan time for next meeting	
		Communication strategies to overcome resistance and promote patient-led change	37
5	Session 2: Continuing the changes (20 minutes) Week 5	Review step-count and walking diary	10, 19
		Encourage progress in increasing walking and achieving step-count goals	12, 13
		Troubleshoot any problems with pedometer or diary	8
		Review target and agree goals for next stage	7, 9, 16
		Barriers and facilitators to increasing PA, overcoming barriers, encouraging support	8, 29
		Pacing and avoiding boom and bust	9, 35
		Check confidence levels, build confidence to make change	18, 29, 36
		Summarise and check patient understanding, plan time for next meeting	
		Communication strategies to overcome resistance and promote patient-led change	37
9	Session 3: Building lasting habits (20 minutes) Week 9	Review step-count and walking diary	10, 19
		Review overall progress over the sessions	11, 17
		Encourage progress in increasing walking and achieving goals	12, 13
		Preparing for setbacks	35
		Building habits: discuss methods of maintaining lasting change, including repetition, if-then rules and support	7, 29, 23, 29, 35
		Setting goals: maintaining current activity or increasing further?	7, 9, 16, 26
		Remind re contact with research assistant in 3–4 weeks	
		Communication strategies to overcome resistance and promote patient-led change	37

1. Provide general information on behaviour-health link; 2. Provide information on consequences to individual; 4. Provide normative information about others’ behaviour; 7. Action planning; 8. Barrier identification; 9. Set graded tasks; 10. Prompt review of behavioural goals; 11. Prompt review of outcome goals; 12. Prompt rewards contingent on effort; 13. Prompt rewards contingent on successful behaviour; 16. Prompt self-monitoring of behaviour; 17. Prompting self-monitoring of behavioural outcome; 18. Prompting focus on past success; 19. Provide feedback on performance; 20. Provide information on when and where to perform the behaviour; 21. Provide instructions on how to perform the behaviour; 23. Teach to use prompts/cues; 26. Prompt practice; 29. Plan social support/social change; 35. Relapse prevention/coping planning; 36.Stress management/emotional control training; 37. Motivational interviewing.

## Study rationale and aims

### Rationale

There is a need for a large, adequately powered primary care trial to test the effect of a pedometer-based walking intervention, with and without nurse PA consultations in inactive adults and older adults. It should include follow-up to 1 year and ensure that adequate numbers of men, older adults and individuals from diverse socio-economic and ethnic backgrounds are included. It should enable the effectiveness of taking part as an individual or as a couple to be estimated. For greatest effect the intervention should use step-goals and diaries and the PA consultations and patient handbook should be based on BCTs, such as those used in the Health Trainer Handbook [57]. To objectively test the interventions’ effectiveness on important public health outcomes, such as time spent in at least moderate intensity activity and time spent sedentary, accelerometer measurement of outcomes should be included. A qualitative assessment is needed to explore the intervention’s acceptability and reasons for dropout and durability of effects. An economic evaluation should be performed alongside the trial and the costs and benefits of the alternatives, modelled beyond the end of the trial.

### Study aims

The main hypotheses to be addressed are: i) does a 3 month pedometer-based walking intervention increase PA in inactive 45–75 year olds at 12 month follow-up; and ii) does providing practice nurse support through PA consultations provide additional benefit. The study will also assess the cost-effectiveness of both interventions and whether or not any effects are modified by age, gender, body mass index or taking part as a couple, and will estimate the effect of the interventions on patient reported outcomes and anthropometric measures.

## Methods/design

This paper was written according to CONSORT reporting guidelines for RCTs of non-pharmacologic treatment [60].

### Trial design

**Figure 1:**
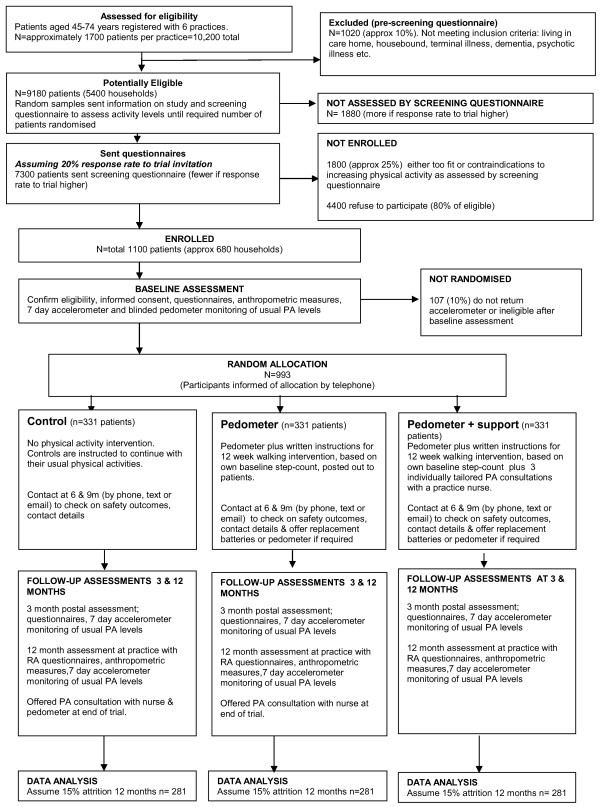
**CONSORT flow diagram for PACE-UP trial.** Detailed legend: CONSORT flow diagram showing participant flow through each stage of the randomized controlled trial (enrolment, intervention allocation, follow-up and data analysis).

A three-arm parallel design cluster RCT with household as the unit of randomisation comparing the following: a control group (usual PA); pedometer and written instructions by post; pedometer and support (written instructions and brief individually tailored PA consultations with a practice nurse). A 1:1:1 allocation will be used. The CONSORT flow diagram summarises the design, procedures and stages (Figure 1) [60].

### Practice and participant recruitment

#### Practice inclusion criteria

South West London general practices with a list size >9,000; giving a commitment to participate over the study duration; having a practice nurse to carry out the PA consultations; and a room for the research assistant to recruit participants and conduct assessments.

#### Practice recruitment

The Primary Care Research Network Greater London will help us to identify potential participant practices within South West London who fit the above practice inclusion criteria. Approaches by mailed invitation, telephone contact with practice managers and personal contact with local general practitioners (GPs) and practice nurses will all be used as necessary to identify practices. We will select six from the list of potentially interested practices to include a range of socio-demographic factors (including targeting some practices in areas with high numbers of ethnic minority patients) and geographical circumstances based on practice postcode index of multiple deprivation scores using national quintiles (at least 1 practice from each quintile). The index of multiple deprivation score includes factors such as distance to services, crime rates and road traffic accident rates, which could influence likelihood of outdoor PA, as well as material deprivation measures [61].

#### Participant inclusion criteria

Patients aged 45–75 years registered at a selected general practice, able to walk outside the home and with no contraindications to increasing their moderate intensity PA levels.

#### Participant exclusion criteria

In order to maximise the benefits of the intervention to individuals and the National Health Service, the trial focusses on less active adults, using a single-item validated questionnaire measure of self-reported PA as a screening question to identify them [51]. Those individuals reporting achieving a minimum of 150 minutes of at least moderate intensity PA weekly [1] will be excluded. Participants found on subsequent baseline accelerometer assessment to be above this PA level will not be excluded, as these patients would be included if this intervention were to be rolled out in primary care. Other exclusions: living in a residential or nursing home; housebound; ≥3 falls in previous year or ≥1 fall in previous year requiring medical attention; terminal illness; dementia or significant cognitive impairment (unable to follow simple instructions); registered blind; new onset chest pain, myocardial infarction, coronary artery bypass graft or angioplasty within the last 3 months; medical or psychiatric condition which the GP considers excludes the patient (e.g., acute systemic illness such as pneumonia, unstable heart failure, unable to move about independently, psychotic illness). Pregnant women will also be excluded.

#### Participant recruitment

The number of patients aged 45–74 years will be recorded at each practice. Practice staff will search practice electronic primary health care records to identify patients aged 45–74, using Read codes supplied by researchers and local care home knowledge to exclude ineligible patients (as above). Initial sampling will include 45–74 year olds, but some individuals will become 75 before randomisation and will still be included. A list of potentially eligible patients will be created and ordered by household, with each household given a unique household identifier. We are aiming to select either individuals or couples in a household, therefore we want to select a maximum of two people per household. If a household with one individual is selected at random, then that individual is selected. If a household has two or more individuals then one individual is selected at random. If there is a second individual in that household with an age difference of 15 years or less, they will also be selected. The approach was based on previous validated work showing that this age difference is an effective way of identifying (married or cohabiting) couples within a household [62]. Initially, the first random sample containing 400 eligible patients will be selected at each practice and the list examined by practice GPs or nurses to ensure trial suitability. Patients in these households will then be mailed an individual trial invitation letter from the practice and the screening question to assess activity levels and a participant information sheet. This will make it clear that if potential participants have any difficulties understanding, speaking or reading English they should bring a family member or friend with them to the research assistant appointment. The participant information sheet will be translated into different languages if practices indicate this to be appropriate. The 400 individuals will be contacted by post in a staggered manner over 2–3 months to avoid overwhelming the research assistants. Reminders will be sent out to non-responders after 6–8 weeks. Further random samples of households will be selected from the list until required numbers have been randomised. On the reply slip, those not wishing to participate will be asked about reasons for declining and their willingness to fill in a health and PA questionnaire, one of the questions on this questionnaire will ask if they would be willing to be interviewed about their reasons for not wanting to participate in the trial. Patients who agree to participate in the trial will be telephoned to arrange a baseline assessment at the practice with the research assistant. Two eligible people within a household will be invited together (or apart if they prefer). Eligibility will be confirmed and informed consent sought at this appointment.

#### Participant selection for the qualitative evaluation

Participant selection for the qualitative evaluation will run parallel to the trial and will focus upon three distinct groups. i) Trial 'non-participants’ who agree to be interviewed, to explore factors influencing their decision not to participate. ii) Purposive samples of four groups of trial participants, after 12-month follow-up (including samples of those who did and did not increase their PA in each of the two intervention arms). The samples will reflect the range of socio-demographic characteristics of participants including ethnicity. iii) All practice nurses (maximum 12 if two per practice) will be invited to participate in a focus group to find out their thoughts about the interventions’ acceptability and use in PA consultations. Interviewing with study participants will continue until no new themes are identified (approximately 55–80 are anticipated, 15–20 for the 'non-participants’ and 10–15 for each of the four groups of trial participants).

### Baseline assessment

The following assessments will be carried out by the research assistant at the patient’s general practice.

i) *Questionnaire measures* – Socio-economic-demographic measures: marital status, ethnic group, occupation, employment, household composition, home ownership. Self-reported PA: modified Zutphen [63]. Health problems and lifestyle factors: self-reported chronic diseases (e.g., heart disease, lung disease, arthritis, depression), disability [64], medication, smoking and alcohol. Patient Reported Outcomes (PROs): exercise self-efficacy [65], anxiety and depression (Hospital Anxiety & Depression Scale [66]), perceived health status (EQ-5D) [67], loneliness [68]. A further self-report questionnaire of 7-day PA recall using the General Practice PA Questionnaire (GPPAQ) [69] and International Physical Activity Questionnaire (IPAQ) [70] will be completed after wearing the PA monitors for 7 days and posted back with them.

ii) *Falls Risk Assessment Tool*[71] – This will be assessed using self-report items and by direct observation of the ability to rise from a chair of knee height without using their arms.

iii) *Anthropometric measures* – Height (measured in bare feet to neared 0.5 cm using a stadiometer); weight (measured to nearest 0.1 kg), body fat, bioimpedance (using Tanita body composition analyser BC-418 MA); and waist and hip circumference (using standard technique and tape measure with clear plastic slider).

iv) *Objective PA assessment* – Measurement of usual PA levels, wearing an accelerometer and a blinded pedometer (Yamax Digiwalker CW200) on a belt over one hip, all day for 7 days, only removing for bathing. A diary is also provided to record what activities are done and how long for. The monitors, belt and diary will be posted back on completion. The Actigraph (GT3X + Manufacturing Technology Inc., Fl. USA) measures vertical accelerations in magnitudes from 0.05–2.0 g sampled at 30 Hz then summed over a selected (5 s) time period, it can record PA continuously for up to 21 days. The output, activity counts per unit of time, distinguishes between different walking speeds and PA intensities, using standard cut-offs [42,43]. The pedometer function on the accelerometer will be used for baseline and outcome measurement of step-counts for the trial. Participants will be offered the option of text messaging to remind them to wear the accelerometer each day and to return it after the 7 days. Once it is returned, the participants receive a £10 gift voucher.

### Randomisation procedure

After all participants in a household have completed the baseline assessment and returned the accelerometer with at least 5 complete days of ≥9 hours / 540 minutes recording, the RA will allocate to the trial groups using an internet randomisation service to ensure independence of the allocation. Participants who do not provide the required data, will be asked to wear the accelerometers for another 7 days or excluded, if this is not possible. To avoid couple contamination, randomisation will be at household level. Block randomisation will be used within practice with random sized blocks to ensure balance in the groups and an even workload for nurses. The research assistant will inform participants by telephone of their group allocation.

### Nature of the complex intervention

**Figure 2:**
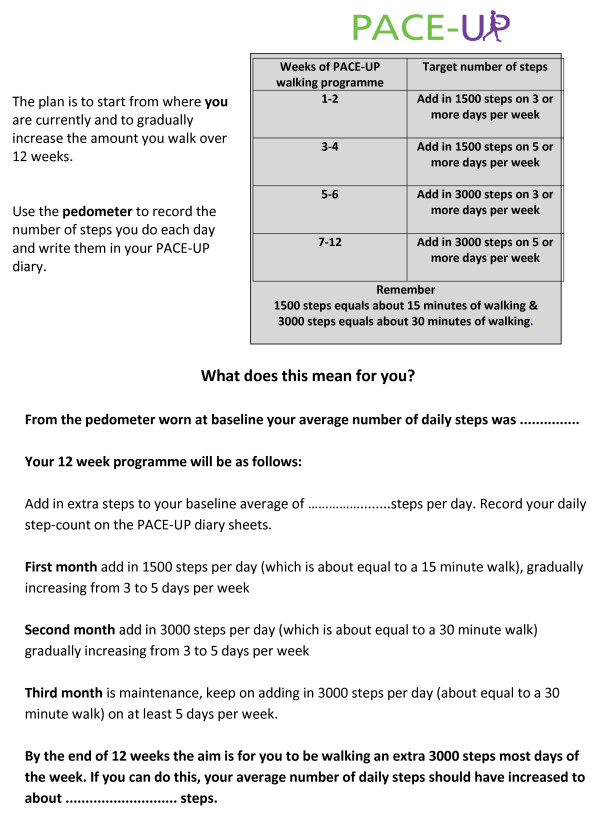
Provides a summary of the PACE-UP walking programme.

Twelve-week pedometer-based walking intervention delivered either by post with written instructions (pedometer group) or delivered in the context of three practice nurse PA consultations (pedometer plus nurse support group). Table 3 provides details of the complex intervention components. (Figure 2)

**Table 3:** Components of the complex intervention for the PACE-UP trial

**Components**	**What was provided**	**Group receipt of components**	**Additional detail on components**
Pedometer	Yamax Digi-Walker (Tokyo, Japan) SW-200 model	Pedometer by post group (sent by post with instructions).	Yamax Digi-Walker is the criterion pedometer with best accuracy [72-74]. The CW200 model is used for baseline target setting, because of 7-day memory of consecutive daily steps, but is bulky to wear and complicated to use. For the intervention groups we are using the SW-200 model, which is compact, cheaper and simpler. It provides direct step-count to participants and requires daily manual recording and re-setting.
		Pedometer plus support group (given by nurse to patients with instructions).	
Patient handbook, walking plan and diary	Patient handbook to support 12-week walking programme. Suggested individualised walking plan (Figure 2). Diary to record weekly PA for 12 weeks (step-count and walks) and whether walking targets have been met each week.	Pedometer by post group (sent by post).	Participants’ baseline average daily step-count (from blinded pedometer assessment) is recorded in the individual’s handbook and diary. Participants have been informed that adding in 3,000 steps/day (approximately equivalent to a 30-minute brisk walk) on 5 or more days weekly to their baseline would help them achieve the recommended PA guidelines, but that this can be built up gradually. The handbook provides advice on the health benefits of at least moderate intensity PA and states that moderate intensity PA makes you warm and a bit breathless and increases your heart rate, but that you should still be able to talk. The handbook and diary provide written advice on maintaining activity, and anticipating and managing setbacks. Table 1 lists the BCTs [58] included in the PACE-UP patient handbook and diary, respectively.
		Pedometer plus support group (given by nurse to patients).	
Practice nurse PA consultations	Three individually tailored PA consultations with the practice nurse. Participants can be seen individually or as a couple.	Pedometer plus support group only.	Session timings, content and planned BCTs [58] (Table 2). Most BCTs overlap with those in the patient handbook and diary to reinforce consultations. The face-to-face nurse consultation allows some additional BCTs to be used; e.g., communication strategies to overcome resistance and promote patient-led change using motivational interviewing techniques and a scale to check confidence levels and build confidence to make change. In the first consultation, the nurse provides the pedometer, patient handbook and diary. The patient’s baseline blinded pedometer average daily step-count is reviewed alongside health and anthropometric data, so that an individual PA plan, tailored to baseline step-count, abilities, health and goals and based on increasing walking and walking speed and other existing PA, can be produced. The nurse shows participants how to use the pedometer and how to record step-counts. Individual tailoring of step-count increase and how fast to increase this is possible. Participants are asked to wear a pedometer and keep daily step-count diary for 4 weeks, until their next appointment. If goals have been achieved new goals can be set, if not, then problems and barriers can be discussed. For couples, both individual goals and opportunities to increase their PA together will be discussed.

### Procedure for control group (usual PA)

The research assistant informs participants that they are in the usual PA group and that they should continue with their usual PA throughout the trial. She/he will thank them for participating and inform them that they will be contacted later to arrange the 3-month postal assessment and the 12-month outcome assessment appointment at the practice, including wearing an accelerometer for 7 days as part of these. He/she will also make contact at 6 and 9 months (by telephone, text, or email according to patient preference) to check on safety outcomes and contact details. On study completion, the control group will be offered a pedometer, diary and written instructions for a 12-week pedometer-based walking programme either by post or as part of a single practice nurse consultation (according to patient preference).

### Procedure for the pedometer-alone group

The research assistant informs participants that they are in the pedometer-alone group and arranges to post out a pedometer, PACE-UP patient handbook and diary with easy to follow written instructions for a 12-week pedometer-based walking programme. This is based on the participant’s own baseline pedometer average daily step-count. The research assistant will telephone 1 week after sending out the pedometer to check that it has arrived safely and is working properly and to offer a replacement pedometer in the event of loss or malfunction during the 12-week intervention. He/she will also check that participants understand the 12-week pedometer-based walking plan and answer any questions. Arrangements for follow-up at 3, 6, 9 and 12 months are as for the control group. In addition, at each follow-up, the research assistant will offer a replacement pedometer or batteries, if required. On study completion, participants in this group will be offered a single practice nurse PA consultation.

### Procedure for the pedometer-plus-nurse-support group

The research assistant informs participants that they are in the pedometer-plus-nurse-support group and arranges a practice nurse appointment for their first PA consultation. Participants can be seen individually or as a couple, for couples both individual goals and opportunities to increase their PA together will be discussed. Arrangements for follow-up at 3, 6, 9 and 12 months are as for the pedometer-alone group.

### Procedure for qualitative study

The qualitative researcher will approach the non-participants and the participants, from both intervention groups, as discussed in qualitative participant recruitment and seek their informed consent for a semi-structured telephone interview. All interviews will be audio-recorded (unless participants do not consent, when contemporaneous field notes will be taken) and transcribed verbatim professionally. Thematic analysis will proceed in parallel with the interviews to enable refinement of the interview guide and purposive sampling according to emerging themes. The qualitative research assistant will also run a focus group with the practice nurses, when all the interventions are completed, this will be audio-recorded and transcribed and subjected to thematic analysis.

### Procedure for the health economics evaluation

The economic evaluation will take the perspective of the National Health Service personal social services and participants and first undertake a trial based analysis. Participant-level resource use data will be collected for equipment (pedometers), face to face or telephone consultations (length of time and frequency), out of pocket expenses (e.g., transport costs), use of support services (number of calls and contacts by post) and for other health service use (e.g., GP attendances, in-patient days, out-patient visits, home visits and services from social services, stays in nursing and residential care). Data will be collected through primary care records, participant questionnaire at 3 and 12 months and monitoring by nurses. Where possible, data collection procedures for the health economics evaluation will be carried out at the same time as those for study effectiveness. Costs that do not vary by use (e.g., development, production and translation of leaflets) will be estimated separately and apportioned to patients within the relevant arm of the trial. Unit costs will be valued using national averages to increase their generalizability. Long-term costs and effects expected to occur beyond the trial will be estimated using Anokye et al.’s model, which accounts for the lifetime risk of developing three conditions associated with PA (coronary heart disease, stroke and type II diabetes) [75].

### Practice nurse training and assessment of fidelity of practice nurse consultations

Practice nurse training in BCTs and in the use of the PACE-UP nurse handbook and PACE-UP patient handbook and diary will be planned with and conducted by experienced trainers in BCTs with primary care and practice nurse training experience (LD and DB) [56]. They will also provide supervision and monitoring to the nurses over the course of the trial, including listening to audio-recordings of a sample of each nurse’s consultations and providing individual feedback. In addition, the Chief Investigator will provide training to the nurses on PA and safety aspects of the trial and the use of pedometers. Nurses will all go on a walk wearing an accelerometer to try out different walking speeds and be shown accelerometer feedback to appreciate the difference between light, moderate and vigorous PA intensities.

The fidelity and quality of the implementation of the intervention will be monitored over time and between different nurses by the following methods: i) analysing the content of a sample of audiorecorded sessions for each nurse by the trainers according to an agreed proforma (to include at least one example of each session and one example of a couple consultation); ii) discussion about consultations during group supervision/training with all the nurses; iii) completion of a checklist of areas covered in each consultation by the nurse; and iv) completion of a nurse patient alliance questionnaire at the end of each patient’s intervention by both the nurse and the patient. The nurse patient alliance questionnaire was drawn up using a modified version of the Working Alliance Inventory [76,77] a validated measure of alliance frequently used in cognitive behavioural therapy based studies, and questions on patient self-efficacy adapted from the SCI Exercise Self-Efficacy Scale [78].

### Assessment of outcomes after 3 and 12 months in the intervention and control groups

#### 3-month postal assessment (interim assessment)

As for baseline assessment (including accelerometer assessment) but there is no anthropometric assessment, and the questionnaire has additional questions about adverse events, injuries and health problems over the last 3 months for all participants and questions on time and financial costs associated with PA and attending nurse appointments for the intervention groups as part of the health economics assessment.

#### 12-month assessment at the patient’s general practice (primary outcome assessment)

As for baseline assessment (including accelerometer assessment) but questionnaire has additional questions about adverse events, including injuries and health problems and use of pedometer over the last 12 months (for pedometer use, slightly different questions depending on group).

Accelerometer data will be downloaded as soon as each accelerometer is returned. Data entry of questionnaire data will occur as soon as possible after data collection at each period. Analysis of outcome data will occur when data on all participants is complete.

### Outcome measures

The primary outcome is change in average daily step-count, measured over 7 days, between baseline and 12 months assessed objectively by accelerometry (Actigraph GT3X + Manufacturing Technology Inc., FL, USA).

Secondary outcomes are:

i) Change in time spent in at least moderate intensity PA and in time spent sedentary between baseline and 12 months, measured over 7 days by accelerometry.

ii) Change in average daily step-count, time spent in at least moderate PA and time spent sedentary measured over 7 days, between baseline and 3 months by accelerometry.

iii) Cost-effectiveness. Incremental cost of the intervention to the National Health Service and incremental cost per change in step-count and per quality adjusted life year.

Other ancillary outcome measures:

i) Change in self-reported PA assessed by GPPAQ and IPAQ.

ii) Change in other patient reported outcomes from the questionnaire (exercise self-efficacy, anxiety, depression, EQ-5D).

iii) Change in anthropometric measurements; weight, BMI, waist circumference, body fat, bioimpedance.

iv) Adverse outcomes; data on falls, injuries, major cardiovascular disease events and deaths will be collected as part of safety monitoring for the trial, through participant and nurse reporting, questionnaires at 3 and 12 months and primary care records after 12 month follow-up.

v) Health service use – number of and diagnoses for all primary care consultations during the 12 months of the trial, as well as any out of hours, A & E, or in-patient attendances that lead to new diagnoses recorded in computerised primary care records, downloaded at the end of the study, given participants’ consent.

#### Qualitative outcomes

There will be a range of outcomes from qualitative interviews and focus groups for non-participants, participants and practice nurses involved in implementing the intervention. We will gain an in-depth understanding of the acceptability and challenges with the interventions for participants and practice nurses, as well as valuable insights into the factors influencing why people opt not to participate in the intervention.

### Sample size

A meta-analysis of a heterogeneous group of short-term intervention studies involving pedometers showed interventions increased steps count per day by 2,500 with a SD of 2,700 [23]. However, a smaller increase in steps of 1,000 per day would lead to worthwhile health gains if this was sustained for 12 months. We also want to be able to demonstrate whether there are differences in the effects achieved by a pedometer intervention alone compared with a pedometer intervention with nurse support. A sample of 217 patients in each of three arms would allow a difference of 1,000 steps per day to be detected between any two arms of the trial with a 90% power at the 1% significance level. This means that we will have sufficient power to adjust for multiple hypothesis testing. However, we plan to randomise households. For men and women the effect of clustering is likely to be small but needs to be taken into account when stratifying by age. Assuming an intra-cluster correlation of 0.5 and an average household size of 1.6 eligible patients we would need to analyse 282 patients per arm. Allowing for approximately 15% attrition, we would need to randomise a total of 993 patients (331 usual PA, 331 pedometer only and 331 pedometer plus nurse support). Six general practices (centres) each recruiting approximately 166 patients will suffice. We will select patients at random to take part until required numbers have been randomised.

### Anticipated recruitment

We anticipate a recruitment rate of 20% amongst those eligible to participate. This estimate is based on pilot work using pedometers and accelerometers in an observational study of older primary care patients, recruitment rate 43% [42] and other studies of PA interventions (including with pedometers) amongst middle-aged and older adults in primary care, where recruitment has been between 6% and 35% [25,79-83]. Even if our recruitment rate were as low as 10%, we would have enough eligible participants (Figure 1).

### Statistical analysis

Analysis and reporting will be in line with CONSORT guidelines, with primary analyses being on an intention-to-treat basis. That is, all participants will be included who have outcome data, regardless of their adherence to the interventions. Sensitivity analyses including all randomised patients will be carried out using multiple imputation to impute PA levels at 12 months for subjects randomised but with no adequate accelerometry data at 12 months; baseline data are available for all subjects by definition. All participants will be included in the primary analysis if they have at least one satisfactory day of accelerometer recording out of 7 days at 12-month follow-up. A satisfactory recording comprises at least 540 minutes (9 hours) of registered time during a day. Adequacy of the randomisation process to achieve balanced groups will be checked by comparing participant characteristics in the three arms (e.g., age, sex, socio-economic group, baseline PA level, health status, body mass index, household size). The same variables will be compared between those who complete follow-up and those who drop out completely, and those who fail to provide a complete set of 5 days data for the primary outcome. Significance tests, either *t*-test or χ^2^ tests, will be used to compare those with complete data and those who have missing outcomes.

### Primary analysis

The primary outcome measure is change in step-count from baseline to 12-month follow-up. Secondary outcome measures which we will also examine are counts per minute, counts per minute of registered time and number of minutes spent in moderate or vigorous PA. These measures are likely to be highly correlated with step count and will be analysed using identical approaches to that for step count. The primary analysis will use all patients with at least 1 day of adequate accelerometry data at 12 months (i.e., complete case analysis). The main outcome will be the change in average daily step-count measured over 7 days between baseline and 12 months. In practice, we will regress average daily step-count at 12 months on average baseline steps per day; this will effectively be measuring change in number of steps over the 12 months.

### Subsidiary analyses

Subsidiary analysis will investigate whether there is any evidence of interaction, that is whether the treatment effect varies by the following factors: age (<60 versus ≥60), gender, socio-economic group, ethnic group, participating as a couple, disability, health status, BMI and exercise self-efficacy. Numbers in each group who have suffered a fracture, falls and injuries, and dropouts will be compared between the groups using logistic regression in STATA, adjusted for clustering.

### Stopping rules

It would be impossible to carry out interim analyses on sufficient patients to decide to stop, so there are no formal statistical stopping rules. If a patient becomes ineligible, the nurse may discontinue the intervention, but all patients will be asked to complete follow-up assessments. Patients can withdraw at any time.

### Procedure for accounting for missing data

Only days with at least 540 minutes of registered time on accelerometer on a given day will be used. Participants in all groups with less than three days satisfactory wear time at follow-up will be asked to wear the accelerometer for an addidional week and the second set of readings used if greater wear time. Participants will only be randomized if they provide at least five such days of accelerometer data at baseline. We will use a mixed effects multilevel linear regression model of daily step count, taking account of day of the week and days since start of measurement to estimate the baseline average daily steps for each subject. The main analysis of effect will include all subjects with at least one satisfactory day of recording at 12 months. We will estimate average daily steps at 12 months for each subject using an identical approach to that at baseline; we will then regress estimated PA level at 12 months on estimated PA level at baseline, age, sex and practice as well as treatment group, while including household as a random effect. In a further sensitivity analysis, we will use multiple imputation to impute values for those with no accelerometer data at 12 months.

### Participant withdrawal

Participants will be free to withdraw from the trial at any time and without giving a reason. Practice nurses can advise discontinuation of the PA intervention if the intervention poses a hazard to the participant. In both cases, information that has already been collected on participants may still be used and they will be asked if they would be prepared to provide any further data on outcomes at 3 months and 12 months (e.g., questionnaire, anthropometric measurements and/or PA monitoring). Withdrawal from the study will not affect the standard of care received by the practice. If participants withdraw before they have been randomised they will be replaced, those withdrawing or being withdrawn after randomisation will not be replaced.

### Adverse event monitoring

#### Notification and reporting of adverse events

A standard operating procedure for the management of adverse events will be in place, so that participants or their relatives, practice staff or researchers can inform the chief investigator of any event. All adverse events reported will be assessed for seriousness, expectedness and causality.

#### Retrospective data collection on adverse events

i) *Questionnaires:* Intervention and control groups will be sent questionnaires at 3 and 12 months that will ask specifically about falls, injuries and exacerbation of any pre-existing conditions in the previous 3- and 12-month periods, respectively.

ii) *Contact with research assistant:* Participants in all three groups will be contacted at 6 and 9 months (by telephone, text or email as preferred by participant) and asked about adverse events since the last contact.

iii) *Computerised primary care records:* In order to be sure that full data on adverse events is collected, informed consent will be sought to collect data from participant records at the end of the study. All consultation data for the 12-month period of the study for each individual will be downloaded from practice computerised records, including all new problems/diagnoses recorded during this period. This will be anonymised before removal from the practice and a researcher who is blind to the intervention or control status of the participants will analyse this data with a standardised proforma recording possible adverse events.

### Ethical and organizational review

The trial has been reviewed and given a favourable opinion by the London Research Ethics Committee (Hampstead) (12/LO/0219). National Health Service Research and Development approval was given initially by Primary Care Trusts and then by Clinical Commissioning Groups in South West London to cover all the practice sites.

## Discussion

The PACE-UP trial is a primary care based PA intervention for inactive 45–75 year olds which seeks to discover if provision of a pedometer by post as part of a 12-week walking programme can increase PA levels at 12 months compared with usual care and whether additional practice nurse PA consultations can increase any effects. It is a pragmatic trial being conducted across several general practices with patients’ own practice nurses, rather than trained researchers or therapists delivering the intervention. The findings will therefore be of direct relevance to UK primary care and other developed countries with similar healthcare provision.

We have taken the following measures in the trial to minimise or avoid bias:

i) *Randomisation:* The Kings College Clinical Trials Unit internet randomisation service will be used to ensure allocation concealment. Randomisation will be at household level to avoid couple contamination (see below).

ii) *Contamination:* Contamination could occur between partners in the same household. This will be minimised by ensuring that if both are recruited they are allocated to the same group. Contamination could also occur in the control 'usual PA’ group if they seek to increase their PA. Participants will be discouraged from buying a pedometer by ensuring that they know that they will receive one, along with instructions on its use and the offer of a PA consultation with their practice nurse and feedback on their individual activity levels at the end of the trial. The 3-month and 12-month assessments will capture information on PA in the usual PA group, including a question at 12 months about whether they have used a pedometer at all in the previous year.

iii) *Blinding and assessment of outcomes:* Participants cannot be blinded to their intervention or control status. The research assistants assessing outcomes will not be blinded to the participants’ intervention status for pragmatic reasons; the study is funded to support only two research assistants to carry out recruitment and follow-up simultaneously at their allocated practices. Appointments for the 3-month and 12-month outcome assessments will be booked in advance according to a protocol, taking into account holidays. However, primary and secondary outcome measures are objectively measured by accelerometry and do not rely on assessor interpretation. Physical measurements will also be assessed objectively (e.g., body weight and body fat measurements using scales with print-out results). Patient reported outcomes will be assessed by validated self-report instruments, minimising researcher bias. The statistician analysing the data will be blind to the treatment allocation of the participants.

The particular challenges that we anticipate in this study are as follows:

i) Low levels of recruitment and possible selection bias, with those who are more physically active being more likely to want to take part. We have a screening question to filter out those who already report recommended PA levels, this should minimize the number who are too active taking part. We are addressing potential low levels of recruitment by recruiting from practices with enough people in the target age range for us to achieve our sample size even if recruitment were as low as 10% of those eligible. In order to estimate response bias we aim to assess self-reported PA and health on those who are not recruited to the trial, but who are willing to fill out a short questionnaire.

ii) Variation in the PA interventions delivered across practices and over time. We have several quality assurance mechanisms in place (including protocols for research assistants who are delivering the postal intervention, and protocols, audio-recording of consultations, group supervision, nurse checklists and patient nurse alliance scales for the nurses delivering the PA consultations) to help us to avoid and monitor these aspects of fidelity.

iii) Losses to follow-up, particularly the control group. We hope to reduce this in the following ways: personal contact with the same research assistant; the offer of a £10 gift voucher when accelerometers are returned; offering controls individual feedback on their activity levels after they complete the trial from their baseline, 3-month and 12-month assessments; and offering a pedometer and 12-week individualized walking programme, either by post or in a single nurse PA consultation, after trial completion.

The findings of this trial will contribute importantly to the development of strategies to address a key global public health challenge, low PA among adults and older adults. Specifically in the UK, an understanding of the role of pedometer-based programmes and nurse support will help guide national policy on promoting PA in primary care. If effective and cost-effective, our interventions could be incorporated into the National Health Service Health Check Programme, which targets patients aged 40–74 years. More widely, our findings will be able to guide international policy and recommendations for increasing PA.

## Trial status

In recruitment phase (recruitment started October 2013 and anticipated to finish November 2013).

## Abbreviations

BCI: Behaviour change intervention; BCTs: Behaviour change techniques; BMI: Body mass index; GP: General practitioner; NICE: National Institute for Health and Clinical Excellence; PA: Physical activity; RCT: Randomised controlled trial; UK: United Kingdom.

## Competing interests

The authors declare that they have no competing interests.

## Authors’ contributions

TH, DC, CV and SK conceived the idea for the study. TH, DC, CV, SK, SS, SI, MU, UE, PW, JF-W and NA participated in the design of the study and developed the research protocol for funding. SK, DC and EL were responsible for the statistical analysis plan, and SK and DC for the sample size calculations. CV and TH designed the qualitative aspects of the study. TH, LD, DB and MU designed the behaviour change intervention, adapted the National Health Service health trainer handbook for the purposes of this trial and designed the patient handbook and patient diary. TH, LD, DB, MU and CF designed and carried out the nurse training. JF-W and NA designed the health economics procedures and data collection tools. CF, EH and RD were involved in compiling patient information and data collection packs and in discussions of any practical changes required to the protocol. EL organised the random samples for each of the practices and data collection and data management plans. TH, JI, SDW, MU and SS were involved in questionnaire development and practice recruitment, selection and training. All the authors have read and approved the final manuscript.

## References

[B1] Department of Health PAHI&P Start Active, Stay Active: A Report on Physical Activity for Health from the Four Home Countries’ Chief Medical Officers 2011 London: UK Department of Health

[B2] World Health Organisation Global Recommendations on Physical Activity for Health 2010 Geneva: WHO 26180873

[B3] Garber CE Blissmer B Deschenes MR Franklin BA Lamonte MJ Lee IM Nieman DC Swain DP American College of Sports Medicine American college of sports medicine position stand: quantity and quality of exercise for developing and maintaining cardiorespiratory, musculoskeletal, and neuromotor fitness in apparently healthy adults: guidance for prescribing exercise Med Sci Sports Exerc 2011 43 1334 1359 10.1249/MSS.0b013e318213fefb 21694556

[B4] O’Donovan G Blazevich AJ Boreham C Cooper AR Crank H Ekelund U Fox KR Gately P Giles-Corti B Gill JM Hamer M McDermott I Murphy M Mutrie N Reilly JJ Saxton JM Stamatakis E The ABC of physical activity for health: a consensus statement from the British association of sport and exercise sciences J Sports Sci 2010 28 573 591 10.1080/02640411003671212 20401789

[B5] Morris JN Hardman AE Walking to health Sports Med 1997 23 306 332 10.2165/00007256-199723050-00004 9181668

[B6] Joint Health Surveys Unit Health Survey for England 2008: Physical Activity & Fitness 2009 London: The NHS Information Centre for Health & Social Care

[B7] Simonsick EM Guralnik JM Volpato S Balfour J Fried LP Just get out the door! Importance of walking outside the home for maintaining mobility: findings from the women’s health and aging study J Am Geriatr Soc 2005 53 198 203 10.1111/j.1532-5415.2005.53103.x 15673341

[B8] National Centre for Social Research Health Survey for England 2004 2005 London: Department of Health

[B9] Sjostrom M Oja P Hagstromer M Smith BJ Bauman A Health-enhancing physical activity across the European union countries: the eurobarometer study J Public Health 2006 14 291 300 10.1007/s10389-006-0031-y

[B10] Centers for Disease Control and Prevention State Indicator Report on physical Activity 2010 Atlanta GA: US Department of Health and Human Services

[B11] Tudor-Locke CE Myers AM Challenges and opportunities for measuring physical activity in sedentary adults Sports Med 2001 31 91 100 10.2165/00007256-200131020-00002 11227981

[B12] Morey MC Sullivan RJ Jr Medical assessment for health advocacy and practical strategies for exercise initiation Am J Prev Med 2003 25 204 208 14552945 10.1016/s0749-3797(03)00180-6

[B13] Cress ME Buchner DM Prohaska T Rimmer J Brown M Macera C Dipietro L Chodzko-Zajko W Best practices for physical activity programs and behavior counseling in older adult populations J Aging Phys Act 2005 13 61 74 15677836 10.1123/japa.13.1.61

[B14] Thompson PD Buchner D Pina IL Balady GJ Williams MA Marcus BH Berra K Blair SN Costa F Franklin B Fletcher GF Gordon NF Pate RR Rodriguez BL Yancey AK Wenger NK American Heart Association Council on Clinical Cardiology Subcommittee on Exercise, Rehabilitation, and Prevention; American Heart Association Council on Nutrition, Physical Activity, and Metabolism Subcommittee on Physical Activity Exercise and physical activity in the prevention and treatment of atherosclerotic cardiovascular disease: a statement from the council on clinical cardiology (subcommittee on exercise, rehabilitation, and prevention) and the council on nutrition, physical activity, and metabolism (subcommittee on physical activity) Circulation 2003 107 3109 3116 10.1161/01.CIR.0000075572.40158.77 12821592

[B15] Hootman JM Macera CA Ainsworth BE Martin M Addy CL Blair SN Association among physical activity level, cardiorespiratory fitness, and risk of musculoskeletal injury Am J Epidemiol 2001 154 251 258 10.1093/aje/154.3.251 11479190

[B16] Ory M Resnick B Jordan PJ Coday M Riebe D Ewing Garber C Pruitt L Bazzarre T Screening, safety, and adverse events in physical activity interventions: collaborative experiences from the behavior change consortium Ann Behav Med 2005 29 20 28 10.1207/s15324796abm2902s_5 15921486

[B17] Hillsdon M Foster C Thorogood M Interventions for promoting physical activity Cochrane Database Syst Rev 2005 1 CD003180 15674903 10.1002/14651858.CD003180.pub2 PMC4164373

[B18] Hillsdon M Foster C Cavill N Crombie H Naidoo B The Effectiveness of Public Health Interventions for Increasing Physical Activity Among Adults: A Review of Reviews 2005 London: HAD

[B19] Hobbs N Godfrey A Lara J Errington L Meyer TD Rochester L White M Mathers JC Sniehotta FF Are behavioral interventions effective in increasing physical activity at 12 to 36 months in adults aged 55 to 70 years? A systematic review and meta-analysis BMC Med 2013 11 75 10.1186/1741-7015-11-75 23506544 PMC3681560

[B20] King AC Rejeski WJ Buchner DM Physical activity interventions targeting older adults: a critical review and recommendations Am J Prev Med 1998 15 316 333 10.1016/S0749-3797(98)00085-3 9838975

[B21] National Institute for Health and Clinical Excellence Behaviour Change at Population, Community and Individual Levels 2007 London: National Institute of Health and Clinical Excellence

[B22] Ogilvie D Foster CE Rothnie H Cavill N Hamilton V Fitzsimons CF Mutrie N Scottish Physical Activity Research Collaboration: Interventions to promote walking: systematic review BMJ 2007 334 1204 10.1136/bmj.39198.722720.BE 17540909 PMC1889976

[B23] Bravata DM Smith-Spangler C Sundaram V Gienger AL Lin N Lewis R Stave CD Olkin I Sirard JR Using pedometers to increase physical activity and improve health: a systematic review JAMA 2007 298 2296 2304 10.1001/jama.298.19.2296 18029834

[B24] Kang M Marshall SJ Barreira TV Lee JO Effect of pedometer-based physical activity interventions: a meta-analysis Res Q Exerc Sport 2009 80 648 655 19791652 10.1080/02701367.2009.10599604

[B25] McMurdo ME Sugden J Argo I Boyle P Johnston DW Sniehotta FF Donnan PT Do pedometers increase physical activity in sedentary older women? A randomized controlled trial J Am Geriatr Soc 2010 58 2099 2106 10.1111/j.1532-5415.2010.03127.x 21054290

[B26] Houle J Doyon O Vadeboncoeur N Turbide G Diaz A Poirier P Innovative program to increase physical activity following an acute coronary syndrome: randomized controlled trial Patient Educ Couns 2011 85 e237 e244 10.1016/j.pec.2011.03.018 21546203

[B27] Yates T Davies M Gorely T Bull F Khunti K Effectiveness of a pragmatic education program designed to promote walking activity in individuals with impaired glucose tolerance: a randomized controlled trial Diabetes Care 2009 32 1404 1410 10.2337/dc09-0130 19602539 PMC2713638

[B28] National Institute for Health and Clinical Excellence Four Commonly Used Methods to Increase Physical Activity: Brief Interventions in Primary Care, Exercise Referral Schemes, Pedometers and Community Based Exercise Programmes for Walking and Cycling. Public Health Intervention Guidance no.2 2006 London: National Institute for Health and Clinical Excellence

[B29] National Institute for Health and Clinical Excellence Walking and Cycling: Local Measures to Promote Walking and Cycling as Forms of Travel or Recreation. NICE Public Health Guidance 41 2012 London: National Institute for Health and Clinical Excellence

[B30] Schneider PL Bassett DR Jr Thompson DL Pronk NP Bielak KM Effects of a 10,000 steps per day goal in overweight adults Am J Health Promot 2006 21 85 89 17152246 10.4278/0890-1171-21.2.85

[B31] Lindberg R Active living: on the road with the 10,000 Steps program J Am Diet Assoc 2000 100 878 879 10.1016/S0002-8223(00)00251-0 10955038

[B32] Croteau KA Richeson NE Farmer BC Jones DB Effect of a pedometer-based intervention on daily step counts of community-dwelling older adults Res Q Exerc Sport 2007 78 401 406 18274211 10.1080/02701367.2007.10599439

[B33] Prochaska JJ Hall SM Humfleet G Munoz RF Reus V Gorecki J Hu D Physical activity as a strategy for maintaining tobacco abstinence: a randomized trial Prev Med 2008 47 215 220 10.1016/j.ypmed.2008.05.006 18572233 PMC2536696

[B34] Tudor-Locke C Craig CL Brown WJ Clemes SA De CK Giles-Corti B Hatano Y Inoue S Matsudo SM Mutrie N Oppert JM Rowe DA Schmidt MD Schofield GM Spence JC Teixeira PJ Tully MA Blair SN How many steps/day are enough? For adults Int J Behav Nutr Phys Act 2011 8 79 10.1186/1479-5868-8-79 21798015 PMC3197470

[B35] Marshall SJ Levy SS Tudor-Locke CE Kolkhorst FW Wooten KM Ji M Macera CA Ainsworth BE Translating physical activity recommendations into a pedometer-based step goal: 3000 steps in 30 minutes Am J Prev Med 2009 36 410 415 10.1016/j.amepre.2009.01.021 19362695

[B36] McKay J Wright A Lowry R Steele K Ryde G Mutrie N Walking on prescription: the utility of a pedometer pack for increasing physical activity in primary care Patient Educ Couns 2009 76 71 76 10.1016/j.pec.2008.11.004 19097843

[B37] Baker G Gray SR Wright A Fitzsimons C Nimmo M Lowry R Mutrie N Scottish Physical Activity Research Collaboration (SPARColl) The effect of a pedometer-based community walking intervention “Walking for Wellbeing in the West” on physical activity levels and health outcomes: a 12-week randomized controlled trial Int J Behav Nutr Phys Act 2008 5 44 10.1186/1479-5868-5-44 18775062 PMC2546435

[B38] Tudor-Locke C Craig CL Aoyagi Y Bell RC Croteau KA De Bourdeaudhuij I Ewald B Gardner AW Hatano Y Lutes LD Matsudo SM Ramirez-Marrero FA Rogers LQ Rowe DA Schmidt MD Tully MA Blair SN How many steps/day are enough? For older adults and special populations Int J Behav Nutr Phys Act 2011 8 80 10.1186/1479-5868-8-80 21798044 PMC3169444

[B39] Macmillan F Fitzsimons C Black K Granat MH Grant MP Grealy M Macdonald H McConnachie A Rowe DA Shaw R Skelton DA Mutrie N West End Walkers 65+: a randomised controlled trial of a primary care-based walking intervention for older adults: study rationale and design BMC Public Health 2011 11 120 10.1186/1471-2458-11-120 21333020 PMC3050749

[B40] Mutrie N Doolin O Fitzsimons CF Grant PM Granat M Grealy M Macdonald H MacMillan F McConnachie A Rowe DA Shaw R Skelton DA Increasing older adults’ walking through primary care: results of a pilot randomized controlled trial Fam Pract 2012 29 6 633 642 10.1093/fampra/cms038 22843637 PMC3501246

[B41] Ekelund U Tingstrom P Kamwendo K Krantz M Nylander E Sjostrom M Bergdahl B The validity of the Computer Science and Applications activity monitor for use in coronary artery disease patients during level walking Clin Physiol Funct Imaging 2002 22 248 253 10.1046/j.1475-097X.2002.00426.x 12402446

[B42] Harris TJ Owen CG Victor CR Adams R Cook DG What factors are associated with physical activity in older people, assessed objectively by accelerometry? Br J Sports Med 2009 43 442 450 10.1136/bjsm.2008.048033 18487253

[B43] Davis MG Fox KR Physical activity patterns assessed by accelerometry in older people Eur J Appl Physiol 2007 100 581 589 10.1007/s00421-006-0320-8 17063361

[B44] Harris TJ Owen CG Victor CR Adams R Ekelund U Cook DG A comparison of questionnaire, accelerometer, and pedometer: measures in older people Med Sci Sports Exerc 2009 41 1392 1402 10.1249/MSS.0b013e31819b3533 19516162

[B45] Tudor-Locke C Ainsworth BE Thompson RW Matthews CE Comparison of pedometer and accelerometer measures of free-living physical activity Med Sci Sports Exerc 2002 34 2045 2051 10.1097/00005768-200212000-00027 12471314

[B46] NICE Public Health Collaborating Centre A Rapid Review of the Effectiveness of Pedometer Interventions to Promote Physical Activity in Adults 2006 London: National Institute for Health and Clinical Excellence

[B47] Talib H Sabirin J Pedometer and Accelerometer in Assessing Physical Activity Among Children and Adolescents 2007 Malaysia: Health Technology Assessment Unit

[B48] NICE Modelling the Cost-Effectiveness of Physical Activity Interventions 2006 London: National Institute for Clinical Excellence

[B49] Shaw R Fenwick E Baker G McAdam C Fitzsimons C Mutrie N 'Pedometers cost buttons’: the feasibility of implementing a pedometer based walking programme within the community BMC Public Health 2011 11 200 10.1186/1471-2458-11-200 21453509 PMC3076275

[B50] Health Checks Programme NHS Putting Prevention First: NHS Health Checks: Vascular Risk Assessment and Management Best Practice Guidelines 2009 London: NHS

[B51] Lawton BA Rose SB Elley CR Dowell AC Fenton A Moyes SA Exercise on prescription for women aged 40–74 recruited through primary care: two year randomised controlled trial BMJ 2008 337 a2509 10.1136/bmj.a2509 19074218 PMC2769033

[B52] Brawley LR Rejeski WJ King AC Promoting physical activity for older adults: the challenges for changing behavior Am J Prev Med 2003 25 172 183 10.1016/S0749-3797(03)00182-X 14552942

[B53] Tai SS Gould M Iliffe S Promoting healthy exercise among older people in general practice: issues in designing and evaluating therapeutic interventions Br J Gen Pract 1997 47 119 122 9101673 PMC1312894

[B54] Goodman C Davies S Tai SS Dinan S Iliffe S Promoting older peoples’ participation in activity, whose responsibility? A case study of the response of health, local government and voluntary organizations J Interprof Care 2007 21 515 528 10.1080/13561820701637204 17891625

[B55] Khan KM Weiler R Blair SN Prescribing exercise in primary care BMJ 2011 343 d4141 10.1136/bmj.d4141 21765112

[B56] David L Using CBT in General Practice: The 10 Minute Consultation 2006 Oxfordshire: Scion

[B57] British Psychological Society Improving Health: Changing Behaviour: NHS Health Trainer Handbook 2008 London: Department of Health

[B58] Michie S Ashford S Sniehotta FF Dombrowski SU Bishop A French DP A refined taxonomy of behaviour change techniques to help people change their physical activity and healthy eating behaviours: the CALO-RE taxonomy Psychol Health 2011 26 1479 1498 10.1080/08870446.2010.540664 21678185

[B59] Harris T Kerry S Victor CR Ekelund U Woodcock A Iliffe S Whincup P Beighton C Ussher M David L Brewin D Adams F Rogers A Cook D Randomised controlled trial of a complex intervention by primary care nurses to increase walking in patients aged 60–74 years: protocol of the PACE-Lift (Pedometer accelerometer consultation evaluation - Lift) trial BMC Public Health 2013 13 5 10.1186/1471-2458-13-5 23289648 PMC3543841

[B60] Boutron I Moher D Altman DG Schulz KF Ravaud P Methods and processes of the CONSORT Group: example of an extension for trials assessing nonpharmacologic treatments Ann Intern Med 2008 148 W60 W66 18283201 10.7326/0003-4819-148-4-200802190-00008-w1

[B61] Noble M McLennan D Wilkinson K Whitworth A Barnes H The English Indices of Deprivation 2007 2008 London: Department of Communities and Local Government

[B62] Shah SM Carey IM Harris T DeWilde S Victor CR Cook DG Do good health and material circumstances protect older people from the increased risk of death after bereavement? Am J Epidemiol 2012 176 689 698 10.1093/aje/kws162 23051600 PMC3472615

[B63] Caspersen CJ Bloemberg BP Saris WH Merritt RK Kromhout D The prevalence of selected physical activities and their relation with coronary heart disease risk factors in elderly men: the Zutphen Study, 1985 Am J Epidemiol 1991 133 1078 1092 2035512 10.1093/oxfordjournals.aje.a115821

[B64] McGee MA Johnson A Kay D The medical research council cognitive functioning and ageing study (MRC CFAS): the description of activities of daily living in five centres in England and Wales Age & Ageing 1998 27 605 613 10.1093/ageing/27.5.605 12683341

[B65] Jette AM Rooks D Lachman M Lin TH Levenson C Heislein D Giorgetti MM Harris BA Home-based resistance training: predictors of participation and adherence Gerontologist 1998 38 412 421 10.1093/geront/38.4.412 9726128

[B66] Zigmond A Snaith R The hospital anxiety and depression scale Acta Psychiatr Scand 1983 67 361 370 10.1111/j.1600-0447.1983.tb09716.x 6880820

[B67] Brooks R EuroQol: the current state of play Health Policy 1996 37 53 72 10.1016/0168-8510(96)00822-6 10158943

[B68] Tunstall J Old and Alone 1957 London: Routledge and Kegan

[B69] Department of Health General Practice Physical Activity Questionnaire 2006 London: UK Department of Health

[B70] Booth ML Assessment of physical activity: an international perspective Res Q Exerc Sport 2000 71 s114 s120 10925833

[B71] Nandy S Parsons S Cryer C Underwood M Rashbrook E Carter Y Eldridge S Close J Skelton D Taylor S Feder G; Falls Prevention Pilot Steering Group: Development and preliminary examination of the predictive validity of the falls risk assessment tool (FRAT) for use in primary care J Public Health (Oxf) 2004 26 138 143 10.1093/pubmed/fdh132 15284315

[B72] Bassett DR Jr Ainsworth BE Leggett SR Mathien CA Main JA Hunter DC Duncan GE Accuracy of five electronic pedometers for measuring distance walked Med Sci Sports Exerc 1996 28 1071 1077 10.1097/00005768-199608000-00019 8871919

[B73] Schneider PL Crouter SE Bassett DR Pedometer measures of free-living physical activity: comparison of 13 models Med Sci Sports Exerc 2004 36 331 335 10.1249/01.MSS.0000113486.60548.E9 14767259

[B74] Le Masurier GC Lee SM Tudor-Locke C Motion sensor accuracy under controlled and free-living conditions Med Sci Sports Exerc 2004 36 905 910 15126728 10.1249/01.mss.0000126777.50188.73

[B75] Anokye N Lord J Fox-Rushby J National Institute for Health and Clinical Excellence Public Health Intervention Guidance Physical Activity Brief Advice for Adults in Primary Care: Component 2 Economic Analysis: Economic Modelling of Brief Advice on Physical Activity for Adults in Primary Care 2012 London: National Institute for Health and Clinical Excellence

[B76] Hovarth AO Greenberg LS Development and validation of the working alliance inventory J Counselling Psychol 1989 36 223 233

[B77] Andrusyna TP Tang TZ DeRubeis RJ Luborsky L The factor structure of the working alliance inventory in cognitive-behavioral therapy J Psychother Pract Res 2001 10 173 178 11402080 PMC3330646

[B78] Kroll T Kehn M Ho P Groah S The SCI Exercise Self-Efficacy Scale (ESES): development and psychometric properties Int J Behav Nutr Phys Act 2007 4 34 10.1186/1479-5868-4-34 17760999 PMC2034591

[B79] Tully MA Cupples ME Chan WS McGlade K Young IS Brisk walking, fitness, and cardiovascular risk: a randomized controlled trial in primary care Prev Med 2005 41 622 628 10.1016/j.ypmed.2004.11.030 15917061

[B80] Little P Dorward M Gralton S Hammerton L Pillinger J White P Moore M McKenna J Payne S A randomised controlled trial of three pragmatic approaches to initiate increased physical activity in sedentary patients with risk factors for cardiovascular disease Br J Gen Pract 2004 54 189 195 15006124 PMC1314829

[B81] Stevens W Hillsdon M Thorogood M McArdle D Cost-effectiveness of a primary care based physical activity intervention in 45–74 year old men and women: a randomised controlled trial Br J Sports Med 1998 32 236 241 10.1136/bjsm.32.3.236 9773174 PMC1756094

[B82] Munro JF Nicholl JP Brazier JE Davey R Cochrane T Cost effectiveness of a community based exercise programme in over 65 year olds: cluster randomised trial J Epidemiol Community Health 2004 58 1004 1010 10.1136/jech.2003.014225 15547060 PMC1732655

[B83] Sugden JA Sniehotta FF Donnan PT Boyle P Johnston DW McMurdo ME The feasibility of using pedometers and brief advice to increase activity in sedentary older women–a pilot study BMC Health Serv Res 2008 8 169 10.1186/1472-6963-8-169 18691392 PMC2527003

